# Effect of antenatal class attendance on fear of childbirth and antenatal stress

**DOI:** 10.11606/s1518-8787.2023057004272

**Published:** 2023-03-29

**Authors:** Selda Yörük, Ayla Acikgoz

**Affiliations:** I Balıkesir Universitesi Faculty of Health Sciences Department of Midwifery Balikesir Türkiye Balıkesir Universitesi. Faculty of Health Sciences. Department of Midwifery. Cagis Campus, Balikesir, Türkiye; II Dokuz Eylul Universitesi Vocational School of Health Services Izmir Türkiye Dokuz Eylul Universitesi. Vocational School of Health Services. Izmir, Türkiye

**Keywords:** Antenatal Care, Antenatal Education, Antenatal Screening, Childbirth Classes, Childbirth Education, Abdominal Pregnancy, Fear, Childbirth, Stress

## Abstract

**OBJECTIVE:**

To assess the effect of attending antenatal classes on fear of childbirth and antenatal stress in nulliparous pregnant women.

**METHODS:**

A total of 133 nulliparous pregnant women participated in the study, which had a quasi-experimental design. Data were collected by a descriptive data form, the Wijma Delivery Expectancy/Experience Questionnaire, and the Antenatal Perceived Stress Inventory (APSI).

**RESULTS:**

A significant correlation was found between antenatal class attendance and having a high schooling level and an intended pregnancy (p < 0.05). The mean fear of childbirth score of pregnant women was 85.50 ± 19.41 before the training and 76.32 ± 20.52 after the training, and the difference between these scores was significant (p < 0.01). Fear of childbirth score were not significantly different between the intervention group and the control group. The mean APSI score of pregnant women in the intervention group was 22.32 ± 6.12 before the training and 21.79 ± 5.97 after the training. However, this difference was not statistically significant (p = 0.70).

**CONCLUSION:**

The fear of childbirth score decreased significantly in the intervention group after the training.

## INTRODUCTION

Fear of childbirth (FOC) is a common problem among pregnant women^1^. The prevalence of FOC in the literature ranges from 3.6% to 29.5%^1 , 2^. The main causes are individual, biological, psychological, and social factors, such as young maternal age, nulliparity, unintended pregnancy, high pre-pregnancy body mass index (BMI), bad obstetric history, poor mental health, anxiety disorders, traumatic birth experience, lack of social support, and low socioeconomic status^1 , 3 , 4^. The cultural belief that childbirth is a dangerous medical event is another significant reason for FOC in women^5 , 6^. The prevalence of FOC in Turkey is 10.7%^7^. FOC is the most important reason for elective cesarean sections, whose rates have increased in the last 15 years^8^. The findings of our cross-cultural study may constitute a methodological advance in the practice and research on FOC for both international and interdisciplinary scholars.

The frequency of FOC is higher among nulliparous women although they have no previous experience on which to base this fear^9^. In nulliparous pregnant women, the stress of being a “new mother” adds to the anxiety of the first childbirth experience^10^. Studies show that pregnant women attending antenatal classes are more adapted to pregnancy, willing and determined to have a normal delivery, and successful in pain management during delivery and breastfeeding after delivery. Moreover, they have less FOC and need less analgesia and anesthesia^11^.

Antenatal class attendance increases women’s self-confidence during childbirth and reduces anxiety and labor pain^16 , 17^. However, although studies show the positive effect of antenatal classes on fear and stress about childbirth and the birth process^14^, some studies pointed that antenatal education has no effect^18 , 19^. The systematic review by Brixval et al. had insufficient evidence to determine whether antenatal education in small classes affects obstetric or psychosocial outcomes^19^. Haapio et al. stated that stress symptoms are the most common manifestation of FOC^20^. Severe FOC has a substantial negative effect on the lives of pregnant women, as they experience anxiety and stress throughout the entire pregnancy^9^. In their systematic review, Dencker et al. showed that stress increases FOC during pregnancy^21^. In pregnant women, anxiety and fear can cause a prolonged active phase of labor and higher labor pain levels^22 , 23^. Thus, nulliparous pregnant women may receive professional antenatal care to manage the pregnancy process and have a positive and safe delivery experience^9^.

Some empirical studies in Turkey assessed the effect of antenatal class attendance on FOC. Most included multiparous women^14^ and few experimental studies assessed FOC, stress, and labor pain in nulliparous pregnant women. In Turkey, antenatal education is provided in antenatal classes, family health centers, public institutions, state and university hospitals, private clinics, and private hospitals. Determining the level and causes of FOC in nulliparous pregnant women should help in the development of the content of antenatal education in Turkey. Moreover, it may be useful to evaluate the effectiveness of antenatal classes for nulliparous pregnant women to help them have a positive delivery experience. Thus, this study aims to assess the effect of antenatal class attendance on FOC and antenatal stress in nulliparous pregnant women.

## METHODS

This non-randomized quasi-experimental study was conducted at a public institution, the largest maternity hospital in a city in Western Turkey, and compared a control group and an intervention group by experimental methods ( Figure ).

**Figure:**
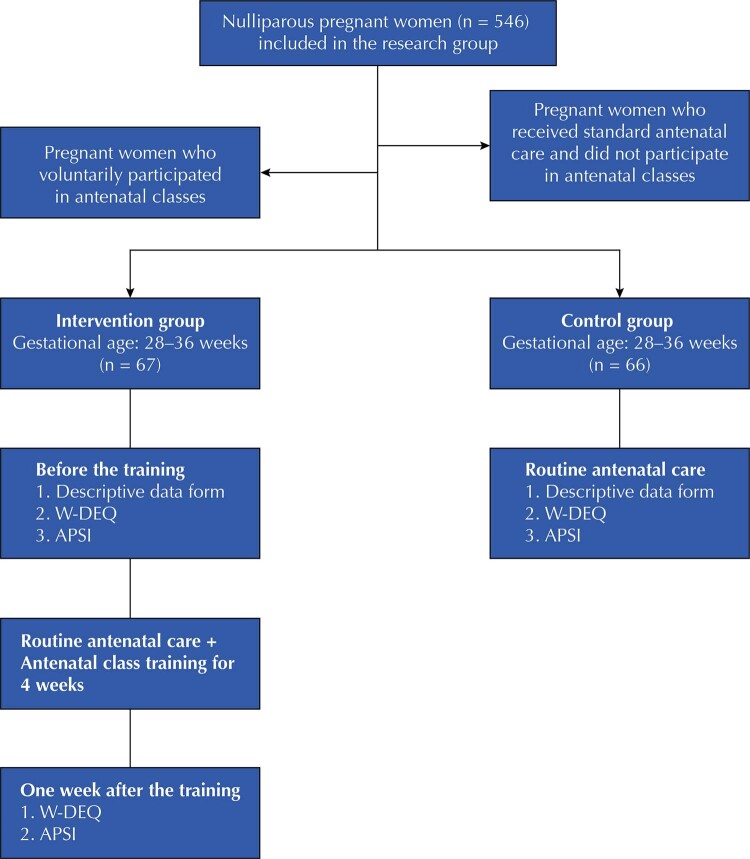
Flowchart of the study.

### The sample

The sample size of the study was estimated according to the sample size of the quasi-experimental research. The study by Serçekuş et al., which included intervention and control groups, found an effect size of 0.98^14^. This effect size is larger than the large effect size (0.80) for Student’s t-test. Thus, the sample size was estimated according to the large effect size expected at the intervention stage of this study using the G-Power program, in order to include at least 42 nulliparous pregnant women in each group, when α = 0.05, power = 0.95, and effect size = 0.80, according to the bidirectional hypothesis. In case of losses, 80 women were initially included in each group. At the end of the study, 67 women were in the intervention group and 66 in the control group.

### Inclusion and Exclusion Criteria

This study included nulliparous 28-to-36-week pregnant women who could communicate effectively, volunteered to participate, were literate, had an intended pregnancy and only one fetus, and, for the intervention group, attended all four antenatal classes. Multiparous pregnant women and women who did not want to participate in the study or had a stillbirth, a chronic disease, a history of miscarriage or abortion, or a risky pregnancy history were excluded from the study.

### Data Collection Tools

Data were collected with a descriptive data form, the Wijma Delivery Expectancy/Experience Questionnaire (W-DEQ), and the Antenatal Perceived Stress Inventory (APSI).

### Descriptive Data Form

This form included questions about the sociodemographic characteristics, pregnancy, and medical history of pregnant women. Sociodemographic questions addressed their current age, age at marriage, region of residence, schooling level, and profession, as well as their spouse’s profession, schooling level, and economic status. Questions about the pregnancy included gestational age, whether the pregnancy was intended, family planning methods used before pregnancy, miscarriage, parity, and health problems experienced during pregnancy. Regarding the medical history , questions addressed surgeries, chronic diseases, infectious diseases, psychiatric disorders, BMI, and substance use. The form was created by researchers using the relevant literature^14 , 18 , 20^. To test its face validity, opinions from three obstetrics experts were obtained and 10 nulliparous women filled in the form in a pilot study.

### Wijma Delivery Expectancy/Experience Questionnaire

The W-DEQ was developed by Wijma et al. to measure FOC^24^. This six-point Likert scale includes 33 items and each question is scored 0 to 5. Its cut-off value is ≥ 85 and higher scores show a higher level of fear^24^. The total score that can be obtained from the scale ranges from 0 to 165. The reliability and validity of its Portuguese version was assessed by Souto et al. The European Portuguese version of the W-DEQ was reliable and effective to measure FOC in both nulliparous and multiparous women^6^. Moreover, this tool can be used to identify and manage women with FOC during the antenatal period^6^. The Cronbach’s α of the scale, adapted to Turkish by Körükcü et al. after conducting validity and reliability tests, was 0.89 ^25^. In this study, the Cronbach’s α of the W-DEQ was 0.91.

### Antenatal Perceived Stress Inventory

The APSI was developed by Razurel et al. to assess perceived stress in the antenatal period^26^. The scale includes 12 items and three subdimensions: medical and obstetric risks/fetal health, psychosocial changes during pregnancy, and prospect of childbirth. The lowest possible score is 12 and the highest is 60. An increase in the total score shows an increase in the level of perceived stress of pregnant women. The Turkish adaptation and validity and reliability tests were performed by Atasever and Sis Celik. The Cronbach’s α of the scale was 0.70^27^. In this study, the Cronbach’s α of the APSI was 0.79.

### Intervention

As the study was quasi-experimental, no randomization was performed. The antenatal training was given by midwives in public hospitals. When pregnant women in the third trimester were admitted for antenatal monitoring, they were directed to voluntary antenatal classes. Antenatal training groups had 10 to 15 people. Classes were held once a week, with four modules per month, and included theory (pregnancy, childbirth and postpartum, and newborn issues), practice (breastfeeding, massage, and breathing), and a visit to the delivery room. Each training module takes an average of three hours. After the training, pregnant women received a certificate of participation. Table 1 presents the contents of each module.

**Table 1:** Contents of the training modules.

Module 1: Reproductive System, Changes during Pregnancy, Breathing, Relaxation, Stretching Exercises
Module 2: Normal Childbirth and Physiology, Drug-Free Coping Methods, Applied Massage Practice
Module 3: Preparing for Childbirth, Preparing Hospital Bag, Ways to Cope with Stress, Family Planning Methods, Breathing, Relaxation, Stretching Exercises, Applied Massage Practice
Module 4: Applied Breastfeeding Techniques and Newborn Care, Postpartum Care, Visit to the Delivery Room

### Control Group

The services provided in the antenatal class were explained to pregnant women in their third trimester who attended to antenatal clinics in public hospitals for routine follow-up and they were directed to participate in these services. Participation in the antenatal class was voluntary. All pregnant women were offered support to participate in the antenatal class, however, only those who wanted to participate received training. The control group included pregnant women who went to the antenatal clinic for routine follow-up and did not want to participate in the antenatal class. To create this group, nulliparous 28-to-36-week pregnant women were randomly selected and their data were collected by a researcher in a face-to-face interview.

### Data Collection

Data were collected from the intervention group before and after the training. The W-DEQ and the APSI were completed in a face-to-face interview before the training and post-training forms were applied one week after the fourth antenatal class in a face-to-face interview. Data from the control group, on the other hand, were collected once using the W-DEQ and the APSI by face-to-face interviews with nulliparous 28-to-36-week pregnant women who received standard antenatal care. Figure shows the flow chart of the study.

### Research Ethics

This study was conducted according to the principles of the Declaration of Helsinki of the World Medical Association (WMA) and ethically approved by the Clinical Research Ethics Committee of Balıkesir University Faculty of Medicine (2019/202). An informed consent was obtained from each participant.

### Statistical Analysis

Statistical analysis was performed using the SPSS 20.0 statistical software. Descriptive statistics were evaluated by determining arithmetic mean, standard deviation, number, and percentage. The Shapiro–Wilk test was used to assess whether data had a normal distribution. Nonparametric tests were used in the analysis, as data did not have a normal distribution.

Sociodemographic characteristics of the intervention and control groups were evaluated by the Mann–Whitney U test and Pearson’s chi-square test. The Mann–Whitney U test was used to compare quantitative variables of both groups (age of pregnant women, weight gained during pregnancy, BMI before pregnancy, gestational age). The chi-square test was used to compare categorical sociodemographic variables (economic status, planned pregnancy, smoking, schooling level, severity of FOC). The Wilcoxon signed-rank test was used to compare the FOC and perceived stress scores of the intervention group before and after the training. The Mann–Whitney U test was used to compare the FOC and perceived stress scores of the intervention and control groups. The level of FOC was grouped according to the FOC cut-off point (< 85, ≥ 85)^24^ and evaluated by McNemar’s chi-square test before and after the training in the intervention group. Pearson’s chi-square test was used to compare the intervention and control groups. Sociodemographic (age, economic status, smoking, schooling level, family type) and obstetric (health problems during pregnancy, planned pregnancy, pre-pregnancy BMI) characteristics and FOC risk factors of pregnant women were evaluated by Pearson’s chi-square test. If the p-value obtained in an analysis was lower than 0.05, the difference was statistically significant.

## RESULTS

The mean age of the intervention group was 27.5 ± 4.63 years (median: 27.5; min.: 19; max.: 35). In total, 91% of this group had secondary or higher education and 91% of their spouses had secondary or higher education. Pre-pregnancy BMI in the intervention group was 24.31 ± 4.66, the mean weight gain during pregnancy was 9.59 ± 6.08 (median: 9.0), and BMI was over 25 in 37.3% of women. A total of 14.9% of this group had more income than expenses, 89.6% had an intended pregnancy, 10.4% smoked, and 17.9% had health problems (bleeding or hyperemesis gravidarum).

The mean age of the control group was 27.89 ± 3.95 years (median: 28.0; min.: 19 max.: 40). A total of 90.9% had secondary or higher education and 78.8% of their spouses had secondary or higher education. Pre-pregnancy BMI of women who did not receive antenatal training was 23.51 ± 3.87. Their mean weight gain during pregnancy was 8.15 ± 4.88 (median: 8.0) and BMI was over 25 in 24.2%. A total of 12.1% of the control group had less income than expenses, 72.7% had an intended pregnancy, 3% smoked, and 15.2% had health problems (bleeding or hyperemesis gravidarum; Table 2 ).

**Table 2:** Sociodemographic and obstetric characteristics of the intervention and control groups

	Intervention (n = 67)		Control (n = 66)		p
	
	Mean ± SD	Median	Mean ± SD	Median	
Age (Mean ± SD and Median)	27.5 ± 4.63	27.5	27.89 ± 3.95	28.0	0.44^a^
Weight gain (Median)	9.59 ± 6.08	9.0	8.15 ± 4.88	8	0.09^a^
Pre-pregnancy BMI (Mean ± SD and Median)	24.31 ± 4.66	23.43	23.51 ± 3.87	22.95	0.37^a^
Gestational age at childbirth (Median [Quartiles])	39 (2)		39 (2)		0.57
Economic status	**n**	**%**	**n**	**%**	
Income equals expenses	53	79.1	48	72.7	0.45
Income is higher than expenses	10	14.9	10	15.2	
Income is lower than expenses	4	6.0	8	12.1	
Pregnancy					
Intended	60	89.6	48	72.7	
Unintended	7	10.4	18	27.3	**0.01**
Smoking					
Yes	7	10.4	2	3.0	
Quit	21	31.3	14	21.2	0.06
No	39	58.2	50	75.8	
Schooling level					
Primary education	6	9.0	6	9.1	0.97
Secondary or higher education	61	91.0	66	90.9	
Spouses’ schooling level					
Primary education	6	9.0	6	9.1	0.97
High school or higher education	61	91.0	66	90.9	
Health problems during pregnancy					
Yes	6	9.0	14	21.2	**0.04**
No	61	91.0	52	78.8	
Fear of childbirth					
Low (≤ 37)	0	0.0	4	6.0	
Moderate (38–65)	11	16.7	15	22.4	
Severe (66–84)	25	37.9	23	34.3	0.34
Clinical (≥ 85)	30	45.5	25	37.3	

SD: standard deviation.

Note: bold shows statistical significance (p < 0.05).

^a^ Mann–Whitney U Test

We found no significant difference between the two groups in terms of mean age, weight gain during pregnancy, BMI, economic status, smoking, schooling level, or health problems during pregnancy. The rates of intended pregnancy and higher spouse’s schooling level were significantly higher in the intervention group (p < 0.05).

Table 2 shows the mean FOC and APSI scores in the intervention and control groups. The FOC score in the intervention group after the training (76.32 ± 20.52) was lower compared with the control group (81.68 ± 16.97). However, this difference was not statistically significant. The mean APSI score was 21.79 ± 5.97 in the intervention group and 22.70 ± 7.03 in the control group. This difference was not statistically significant. Moreover, we found no significant difference in the percentage of women with a FOC score ≥ 85 between the intervention and control groups.

The mean FOC score of women who received antenatal training was 85.50 ± 19.41 before the training and 76.32 ± 20.52 after the training. The difference was statistically significant (p < 0.01). Although the APSI score in the intervention group decreased after the training (from 22.32 ± 6.12 to 21.79 ± 5.97), this difference was not statistically significant (p = 0.70; Table 3 ).

**Table 3:** Relationship between fear of childbirth and perceived stress scores of the control and intervention groups before and after the training

		Intervention group (Before the training) (n = 67)	Intervention group (After the training) (n = 67)	p	Control group (n = 66)	Intervention group (After the training) (n = 67)	p
Fear of childbirth	Mean ± SD	85.50 ± 19.41 (80.77–90.24)	76.32 ± 20.52 (71.32–81.33)	0.01^a^	81.68 ± 16.97 (77.50–85.85)	76.32 ± 20.52 (71.32–81.33)	0.42^b^
	Median	85.00	81.00		82.0	81.00	
Perceived stress	Mean ± SD (95%CI)	22.32 ± 6.12 (20.18–24.45)	21.79 ± 5.97 (19.70–23.88)	0.70^a^	22.70 ± 7.03 (20.24–25.16)	21.79 ± 5.97 (19.70–23.88)	0.58^b^
	Median	21.50	20.00		20.50	20.00	
Fear of childbirth							
< 85	n (%)	33 (49.3)	42 (62.7)		36 (54.5)	42 (62.7)	
≥ 85	n (%)	34 (50.7)	25 (37.3)	0.15^c^	30 (45.5)	25 (37.3)	0.34^d^

SD: standard deviation.

Note: bold shows statistical significance (p < 0.05).

^a^ Wilcoxon signed-rank test.

^b^ Mann–Whitney U test.

^c^ McNemar’s chi-square test.

^d^ Pearson’s chi-square test.

We found no relationship between sociodemographic characteristics of pregnant women and the level of FOC ( Table 4 ).

**Table 4:** Comparison of level of fear of childbirth according to maternal characteristics (n = 133)

Variable	FOC				

	< 85		≥ 85		p*
	
	n	%	n	%	
Age					
18–22	10	55.6	8	44.4	0.89
23–34	55	50.9	53	49.1	
≥ 35	4	51.1	3	42.9	
Economic status					
Income equals expenses	52	51.5	49	48.5	0.88
Income is higher than expenses	10	50.0	10	50.0	
Income is lower than expenses	7	58.3	5	41.7	
Pregnancy					
Intended	53	49.1	55	50.9	0.17
Unintended	16	64.0	9	36.0	
Smoking					
Yes	2	22.2	7	77.8	0.06
No/Quit	67	54.0	57	46.0	
Schooling level					
Primary education	8	66.7	4	33.3	0.28
Secondary or higher education	61	50.4	60	49.6	
Spouses’ schooling level					
Primary education	15	65.2	8	34.8	0.05
Secondary or higher education	54	49.1	56	50.9	
Health problems during pregnancy					
Yes	49	48.5	52	51.5	0.16
No	20	62.5	12	37.5	
BMI (Pre-pregnancy)					
<25	48	52.2	44	47.8	0.91
≥25	21	51.2	20	48.8	
Family type					
Extended family	7	87.5	1	12.5	0.06
Nuclear family	62	49.6	63	50.4	

FOC: fear of childbirth.

^a^ Pearson’s chi-square test.

## DISCUSSION

We aimed to assess how hospital-based planned antenatal training for nulliparous women affects the relationship between FOC, stress, and labor pain. Our study evaluated FOC and stress levels of nulliparous women who attended or not antenatal training and found that antenatal training was effective in reducing FOC in the intervention group, but it had no effect on stress during pregnancy. Moreover, we found no significant difference in the mean FOC and stress scores between the intervention and control groups. In this study, among the sociodemographic and obstetric characteristics of participants, intended pregnancies and high spouse’s schooling level were factors significantly associated with participating in the antenatal class.

An important factor that affects the preparation for pregnancy and childbirth is intended pregnancy. Several studies show that the rates of receiving antenatal care and attending antenatal classes were higher among women with intended pregnancies. Abame et al. found that unintended pregnancies are associated with less use of antenatal care, which supports our findings^28^. However, in contrast to our findings, Brixval et al. and Karabulut et al. found that the rates of intended pregnancy were similar in the groups attending and not attending antenatal classes^19 , 29^. The socioeconomic status was higher among women in the intervention group compared with the control group. In the study by Gluck, educated nulliparous pregnant women with a high income received more antenatal training. Moreover, pregnant women with a high socioeconomic status had higher awareness in attending antenatal classes^30^. Women with intended pregnancies are probably more willing to take steps to reduce risks associated with pregnancy and more knowledgeable and skilled in baby care. We believe that this motivated women with intended pregnancies to attend antenatal classes.

Education is the most important socioeconomic characteristic that positively affects health behaviors in low-income countries. As the spouses’ schooling level increases, they tend to become more sensitive to health issues and have greater knowledge about health needs. A study conducted in 37 developing Asian and African countries showed a significantly positive relationship between higher schooling levels and receiving antenatal care^31^.

In this study, the W-DEQ scores of the intervention and control groups were higher before the training. Half of the pregnant women in the intervention group and one-third of the control group had W-DEQ scores at a clinical level (≥ 85)^24^. The W-DEQ scores of both groups before and after the training were higher compared with studies conducted in different parts of Turkey^14 , 29^. Our study did not evaluate psychological factors. The literature shows that FOC was higher in women with a history of psychiatric disorders, such as childhood abuse, post-traumatic stress disorder, and depression^32^.

In our study, we found no significant difference between the W-DEQ scores of pregnant women in the intervention and control groups before and after the training. However, they significantly decreased after the training in the intervention group. Thus, antenatal training was effective in reducing FOC scores in the intervention group. In their meta-analysis, Hosseini et al. showed that antenatal training was associated with an approximately three-fold decrease in FOC^33^. Our study showed that providing planned antenatal training to nulliparous pregnant women with a high FOC score was effective in reducing FOC. Experimental intervention studies in the literature showed a significant decrease in FOC after the training^14 , 29 , 30^. In the randomized controlled trial (RCT) by Haapio et al., which compared nulliparous women, FOC was significantly higher in the control group^34^. In this study, both before and after the training, the APSI scores of the intervention group were similar to those of the control group during pregnancy. Moreover, we found no significant difference between antenatal training and stress. Similarly to our findings, in their randomized controlled study with a very large sample, Brixval et al. found that the perceived stress scores were similar in pregnant women receiving antenatal training in a small class and women receiving standard training^19^. In the randomized controlled study by Çankaya and Şimşek, antenatal education significantly reduced stress in primiparous pregnant women; the content of the training included information about psychological preparation for pregnancy and childbirth^35^. In the study conducted by Koushade et al., antenatal education in small classes produced a slightly favorable effect on stress levels within six months after childbirth^15^. One of the factors responsible for reducing stress in antenatal education is the inclusion of content on psychology and psychological interventions, such as psycho-education^35 , 36^. However, the effect of antenatal education on antenatal stress is limited to RCTs with a small sample^15^. As an additional important factor, individual and environmental characteristics are associated with stress, but we did not evaluate these factors in this study. The lifestyle and habits of pregnant women^37^ and their sociodemographic and obstetric characteristics^38^, including primigravity, low social support, high maternal–fetal attachment, and high risk of antenatal depression^39^, are “psychosocial stress” factors associated with antenatal stress in the literature. These stress-inducing factors are serious stressors for pregnant women and can not be changed, and the effect of antenatal education on reducing stress due to these factors may be very low.

Our study is an important contribution, as it is an experimental study with a large number of nulliparous women. However, the study has some limitations. The fact that not all pregnant women were in the same trimester may have produced confounding effects on FOC and stress levels. We also could not determine whether nulliparous pregnant women with higher FOC were more willing to receive antenatal training. The frequency of FOC and risk factors among community-based nulliparous pregnant women should be determined qualitatively and quantitatively. Thus, data from this study cannot be generalized. Moreover, we could not determine socioeconomic, environmental, and cultural factors affecting the FOC scores in the intervention group. However, this study will provide information about FOC in nulliparous pregnant women for future qualitative studies.

Our study showed that the FOC score of pregnant women in the intervention group was higher and antenatal class attendance was related to both intended pregnancy and high spouses’ schooling levels. Moreover, the FOC scores decreased significantly after the training in the intervention group. However, we found neither a significant difference between the FOC scores in the intervention and control groups nor a significant relationship between antenatal class attendance and antenatal stress or maternal characteristics and the FOC score.
